# Detection of Advanced Glycosylation End Products in the Cornea Based on Molecular Fluorescence and Machine Learning

**DOI:** 10.3390/bios13020170

**Published:** 2023-01-21

**Authors:** Jianming Zhu, Sifeng Lian, Haochen Zhong, Ruiyang Sun, Zhenbang Xiao, Hua Li

**Affiliations:** 1 School of Life and Environmental Sciences, Guilin University of Electronic Technology, Guilin 541004, China; 2 Guangxi Key Laboratory of Automatic Detecting Technology and Instruments, Guilin University of Electronic Technology, Guilin 541004, China

**Keywords:** advanced glycation end products, cornea, correlation analysis, machine learning, regression analysis, noninvasive detection

## Abstract

Advanced glycosylation end products (AGEs) are continuously produced and accumulated in the bodies of diabetic patients. To effectively predict disease trends in diabetic patients, a corneal fluorescence detection device was designed based on the autofluorescence properties of AGEs, and corneal fluorescence measurements were performed on 83 volunteers. Multiple linear regression (MLR), extreme gradient boosting (XGBoost), support vector regression (SVR), and back-propagation neural network (BPNN) were used to predict the human AGE content. Physiological parameters which may affect corneal AGE content were collected for a correlation analysis to select the features that had a strong correlation with the corneal concentration of AGEs to participate in modeling. By comparing the predictive effects of the four models in the two cases of a single-input feature and a multi-input feature, it was found that the model with the single-input feature had a better predictive effect. In this case, corneal AGE content was predicted by a single-input SVR model, with the average error rate (AER), mean square error (MSE), and determination coefficient R-squared (*R^2^*) of the SVR model calculated as 2.43%, 0.026, and 0.932, respectively. These results proved the potential of our method and device for noninvasive detection of the concentration of AGEs in the cornea.

## 1. Introduction

An important reason for the production of advanced glycosylation end products (AGEs) is the long-term persistence of hyperglycemia in the human environment, when the persistent hyperglycemic state in the body accelerates the Maillard reaction between carbonyl compounds and amino compounds, eventually forming a class of end products that the body cannot metabolize [1,2,3]. AGEs increase slowly with age in normal subjects, while they accumulate dramatically in diabetic patients. Due to their nonmetabolizable properties, the concentration of AGEs in the human body can indirectly reflect the long-term glycemic control of diabetic patients [4,5]. Furthermore, the detection of AGEs can predict the probability of future diabetic complications in patients and provide a basis for assessing the degree of diabetes [6,7].

Currently, AGEs are mainly detected by methods such as the enzyme-linked immunosorbent assay (ELISA), chromatographic analysis, and the molecular fluorescence assay (MFA) [8,9]. Compared with ELISA, which is more widely used and more accurate, MFA has the advantage of being noninvasive and simple to perform [10].

The MFA method for the determination of AGEs is based on the fluorescent properties of AGEs and is achieved by fitting the relationship between the fluorescent intensity at a specific wavelength and the concentration of AGEs. Noninvasive detection of AGEs has become an important research direction in the biomedical field in recent years [11,12,13]. Hull et al. [14] developed a skin AGEs meter in which a certain bandwidth of ultraviolet (UV) light was applied to excite the skin, and thus the concentration of AGEs was measured by observing the intensity of autofluorescence. However, this method is not perfect. On the one hand, skin pigmentation severely affects the intensity of autofluorescence. On the other hand, the short period of metabolism makes it difficult to reflect the long-term accumulation level of AGEs. Van Best et al. [15] designed an instrument using a 460 nm excitation light source to measure corneal autofluorescence, demonstrating the existence of corneal fluorescence and the safety of the test. Compared with the device constructed by Van Best et al., our instrument adopted an optical fiber conductive structure that reduces the interference of light and uses excitation light at 370 nm. At the same time, our device has higher detection accuracy and conforms to the latest international standards, ensuring safety during measurement.

In this study, we aimed to measure autofluorescence in corneas with good optical properties and develop a prediction model for AGE content [16]. An instrument designed for determining the AGE content in corneas based on measurements of the fluorescence intensity measurement was successfully constructed. In addition, four commonly used algorithms, namely multiple linear regression (MLR), extreme gradient boosting (XGBoost) regression, support vector regression (SVR), and back-propagation neural network (BPNN), were used to develop regression models for predicting the content of AGEs. The functions in the sklearn library were used for training the model. The training set and test set were divided appropriately. The feasibility was experimentally verified.

## 2. Principles and Device

### 2.1. Principles of Fluorescence Quantification of AGEs

AGEs are compounds with multiple structures, and some of them have fluorescent properties. For example, pentosidine and tyrosine have strong fluorescence characteristics. The former are excited at approximately 335 nm and emit at 385 nm, and the latter are excited at 275 nm with a fluorescence emission peak at 300 nm. We chose to assay the concentration of glycosylated collagen, a substance that correlates with the total concentration of AGEs. The excitation wavelength of glycosylated collagen is 370 nm and the emission wavelength is 440 nm [17,18]. Therefore, the excitation light wavelength was 370 nm, and the emission wavelength at 440 nm was detected.

The intensity of fluorescence emitted by a fluorescent substance is proportional to its absorbed light intensity, as shown in Equation (1)

(1) $$F=K^{\prime }\left(I_{0}-I\right)$$

where *F* represents the intensity of fluorescence emitted by the excitation of the fluorescent substance, *I_0_* represents the incident intensity of the specific wavelength of the excitation light used when irradiating the fluorescent substance, *I* represents the transmitted light’s intensity after the excitation light irradiates the fluorescent substance, and *K*′ is the fluorescence quantum yield of the substance being tested [19].

According to the Lambert–Beer law, as shown in Equation (2):

(2) $$I=I_{0}10^{-\varepsilon bc}$$

In the equation above, ε indicates the absorption coefficient of the fluorescent substance to the excitation light affected by the concentration of the substance and other factors, *b* indicates the distance passed when the excitation light irradiates the fluorescent substance, which is related to the thickness of the substance, and *c* indicates the concentration of the fluorescent substance in the solution. According to Equations (1) and (2), we obtained the following.

(3) $$F=K^{\prime }I_{0}\left(1-10^{-\varepsilon bc}\right)=K^{\prime }I_{0}\left(1-e^{-2.303\varepsilon bc}\right)$$

Assuming that the values of *c* and $K=2.303K^{\prime }I_{0}\varepsilon bc$ are small, when εbc≤0.05, $e^{-2.303\varepsilon bc}$ ≈ 1, Equation (3) can be reduced to:

(4) $$F=2.303K^{\prime }I_{0}\varepsilon bc=Kc$$

In Equation (4), $K=2.303K^{\prime }I_{0}\varepsilon b$ is a constant and *c* is the concentration of the substance. From Equation (4), it can be seen that when *c* is small, *c* and *F* can achieve an ideal linear function, which means that once the fluorescence intensity has been measured, *c* can be determined. This relationship can be used as a basis for the quantitative analysis of fluorescent substances [20].

### 2.2. Composition of the System

**Figure 1:**
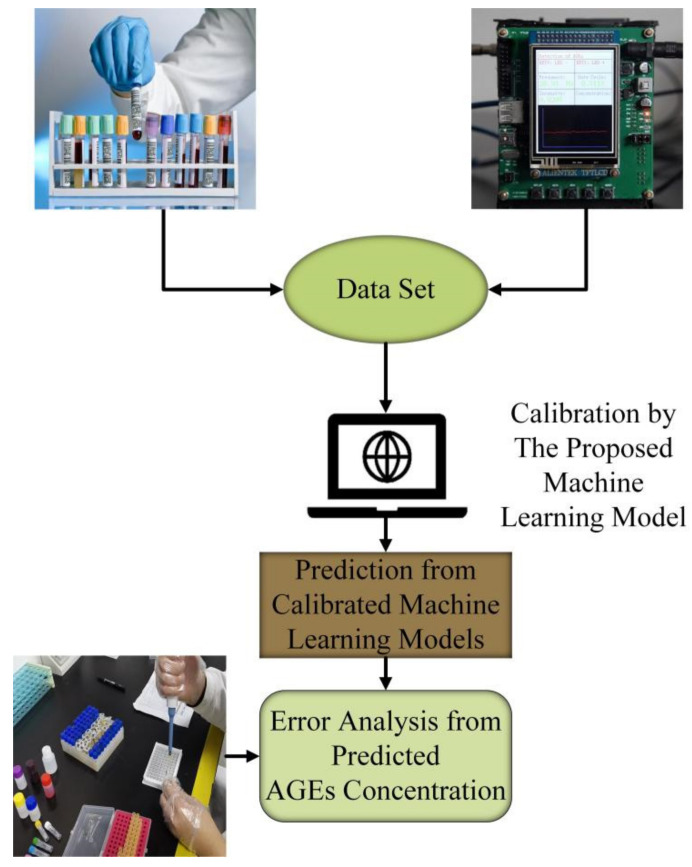
Working process of the proposed device.

We developed a portable fluorescence detection device to measure the autofluorescence in the cornea. The detection system mainly consists of a light source system, a fluorescence acquisition system, and a prediction system. The working process of the system is shown in Figure 1.

### 2.3. Corneal Fluorometry Device

**Figure 2:**
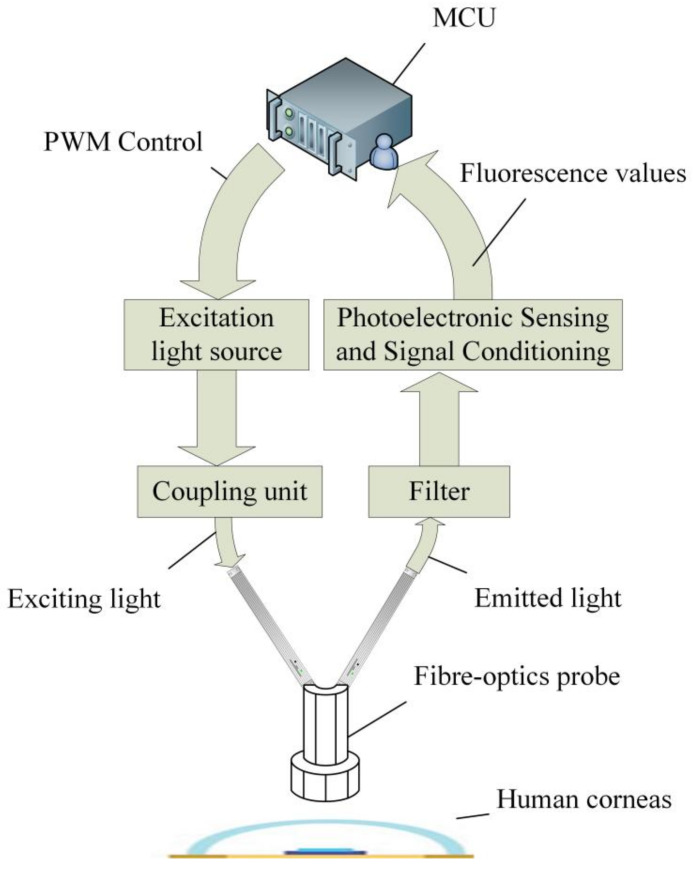
Structure of the device.

In this study, we designed a corneal AGE detector based on human autofluorescence properties. The device is composed of two main parts: an excitation light source system and a fluorescence acquisition system. The micro control unit (MCU) controls the LED driver unit to generate a sTable 370 nm excitation light. The excitation light is coupled to a Y-type 12+1 fiber for transmission. The procedure of detection is to excite and collect the emitted fluorescence by irradiating the cornea, which is then detected using a Y-type 12+1 fiber coupled to a photoconverter. To reduce interference, narrow band filters were added to the excitation and emission light paths. A photoconverter with a high-sensitivity programmable photoelectric sensor was sourced from American Texas Advanced Optoelectronic Solutions (TAOS). To ensure the stable operation of the system, the system incorporated an independent temperature control unit and a high-power heat sink to ensure that the device was not affected by temperature variations. In order to maintain the optimal distance between the equipment and the eyes, the volunteers’ heads were fixed, and the distance between the optical fiber probe and the eyes was adjusted within a reasonable range. On the basis of these principles, the fluorescence intensity of the cornea under a specific wavelength of excitation light could be measured, and thus the AGE content could be inferred. A schematic diagram of the device is shown in Figure 2.

**Figure 3:**
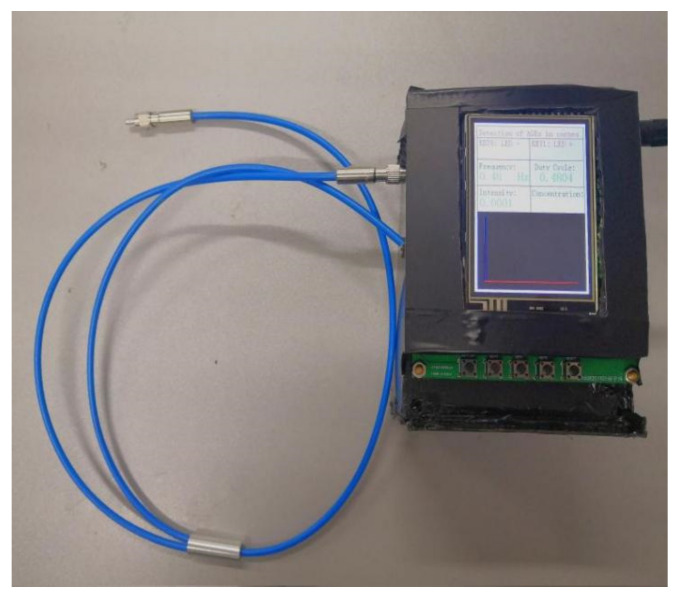
Physical view of the corneal fluorescence measurement device.

Figure 3 shows a physical diagram of the corneal fluorescence measurement device. It mainly consists of a Y-type 12+1 fiberoptic on the left-hand side and the main unit of the device on the right-hand side.

## 3. Methods and Feature Selection

### 3.1. Experimental Procedure

In this study, we recruited 83 volunteers from a hospital in Ganzhou City, including 67 volunteers with Type 2 diabetes. There were 39 males and 28 females in the diseased group, and 8 males and 8 females in the healthy group. All volunteers were informed about the experiment and the possible risks they faced. The experiments met the ethical standards and were conducted after the volunteers signed an informed consent form.

The steps of the experiment were as follows. Each volunteer was tested in the morning on an empty stomach, and physiological data such as height, weight, duration of illness, blood pressure, heart rate, fasting blood glucose (GLU), and glycated hemoglobin (HbA1c) were obtained from the volunteers with the help of medical personnel. After each volunteer in the hospital had their blood sample drawn, they were placed in a darkened space to avoid disturbance from environmental factors. Before starting the measurement, the volunteer was asked to sit comfortably in a chair and wait for a stable state so we could perform the corneal fluorescence measurement using the corneal fluorescence measurement device. When the excitation light source was relatively stable, the process of collecting the data on the AGEs’ fluorescence intensity in the corneas was started. Patients were tested three times separately, with a 5-min interval between each test. Three consecutive measurements were taken for a single test and averaged, with the final result being the results of the three tests averaged again. In addition, ELISA kits (96T, Jiubang Bio, China) were used to measure AGEs in volunteers’ sera separately in the hospital’s laboratory department. These professional operations were performed by medical personnel. The physiological parameters and the data on the fluorescence intensity of corneal AGEs are shown in Table 1.

### 3.2. Selection of Features

**Figure 4:**
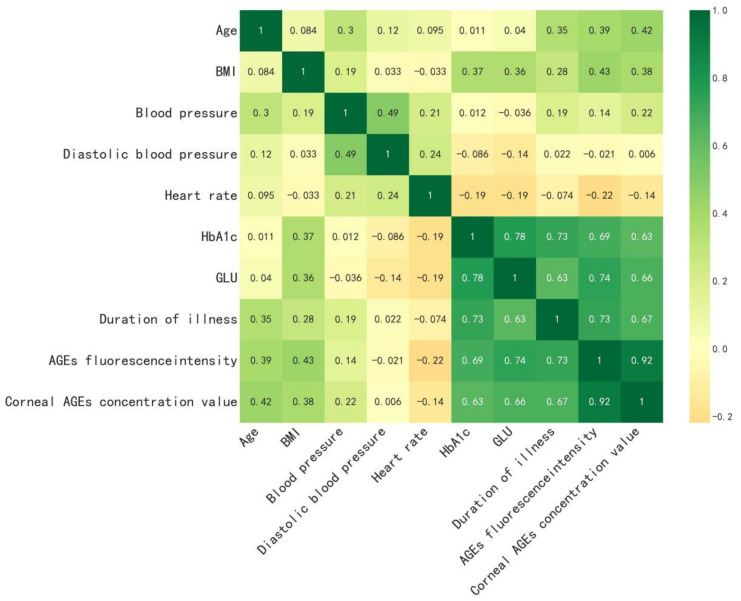
Correlation matrix heatmap.

The alternative modeling parameters were professionally tested in the hospital, and the collected alternative modeling parameters (weight, age, duration of disease, blood pressure, heart rate, HbA1c, GLU, and corneal AGEs’ fluorescence intensity values) were analyzed for correlation. Pearson’s correlation coefficient was used to calculate the correlation matrix of the data, and a heat map of the data correlation analysis was drawn to easily see the correlation of the data, as shown in Figure 4. In Figure 4, we can easily see that the concentration of corneal AGEs was strongly correlated with corneal AGEs’ fluorescence intensity values; moderately correlated with the duration of the disease, GLU, and HbA1c; weakly correlated with age and BMI; and not correlated with blood pressure and heart rate. The weakly correlated features did not improve the accuracy of the model. Since the feature of corneal AGEs’ fluorescence intensity value had a strong correlation with the target and with three features (duration of the disease, HbA1c, and fasting GLU), we decided to use the four input features of corneal AGEs’ fluorescence intensity, duration of the disease, HbA1c, and fasting GLU for further modeling.

For the processing of the missing values of the input features, since the samples collected from the experiments in this study were only missing the disease duration for three patients, the average of the remaining data of this feature was taken for filling the missing data to ensure the sample size and data reliability [21].

## 4. Machine Learning Model for Predicting the Level of AGEs

### 4.1. MLR Model

**Figure 5:**
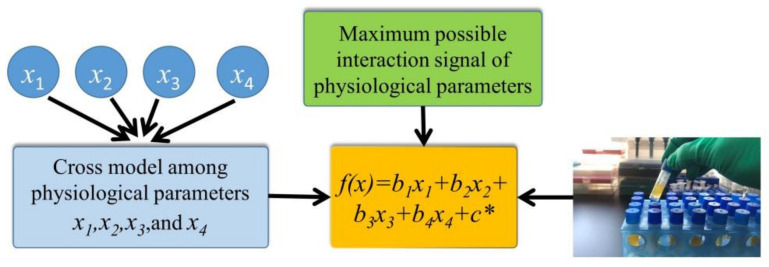
The MLR model used for prediction.

MLR is a method of data analysis that looks for the intrinsic links that exist between variables [22]. For the experiments in this study, the functional relationships for the cases with a single input and with four inputs are as follows:

(5) $$f_{1}\left(x\right)=a_{1}x_{1}+c$$

(6) $$f_{4}\left(x\right)=b_{1}x_{1}+b_{2}x_{2}+b_{3}x_{3}+b_{4}x_{4}+c^{*}$$

where *x*_1_*, x*_2_*, x*_3_*,* and *x*_4_ represent the four features of corneal AGEs’ fluorescence intensity, disease duration, GLU, and HbA1c, respectively; *a*_1_ is the feature coefficient of the single-input feature model; *b*_1_–*b*_4_ are the individual feature coefficients of the four-input feature model; *c* and c* are the intercept terms of both; and *f*_1_(*x*) and *f*_4_(*x*) represent the concentration of corneal AGEs. A general diagram of the four-input feature model is shown in Figure 5.

### 4.2. XGBoost Regression Model

XGBoost is a tree-based integration algorithm proposed by Chen et al. [23]. It models by using multiple learners and has higher accuracy than a single learner. XGBoost uses the idea of boosting, which generates subtrees in a parallel manner, and finally sums up the predictions of multiple decision trees to obtain the final prediction result. It is different from the gradient boosting decision tree (GBDT) algorithm, which also uses the idea of boosting. In GBDT, each time the next weak learner is generated, the gradient of the loss function is used as the learning target, which is equivalent to optimizing with the gradient descent to approximate the minimum of the loss function, even if the loss function is zero and the final learner is as close to the true result as possible [24,25]. However, in XGBoost, the difference of the second-order Taylor expansion of the loss function was taken as the learning objective, which is equivalent to using Newton’s method of optimization to approximate the minimum of the loss function, which means making the loss function equal to zero. The model’s structure can be represented as follows:

(7) $$\hat{y}=\overset{L}{\underset{l}{\sum }}f\left(x_{j}\right),f_{l}\in F$$

where $\hat{y}$ is the final prediction of the model, *L* denotes the number of combined decision trees as the number of trees to be reconciled, *f_l_* is the *l*th tree, *x_j_* means the *j*th input sample, and *F* means the set of all tree models. The objective function and canonical terms used in the model are as follows:

(8) $$Obj^{\left(t\right)}=\overset{n}{\underset{j=1}{\sum }}loss\left({\hat{y_{j}}}^{\left(t-1\right)},y_{j}+f_{t}\left(x_{j}\right)\right)+\Omega \left(f_{t}\right)$$

**Figure 6:**
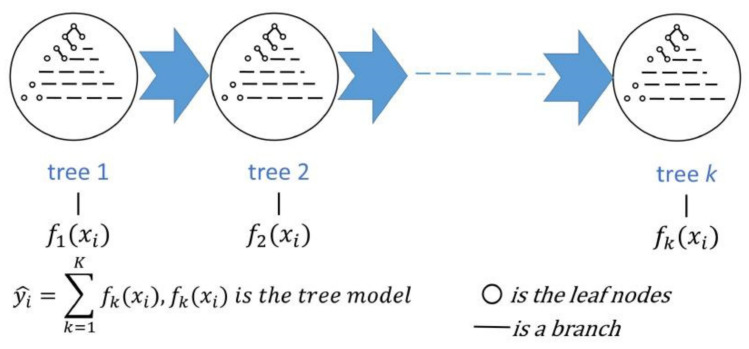
The XGBoost model used for prediction.

In Equation (8), $loss\left({\hat{y_{j}}}^{\left(t-1\right)},y_{j}+f_{t}\left(x_{j}\right)\right)$ is the loss function, $\Omega \left(f_{t}\right)$ is the canonical term, $\Omega \left(f_{t}\right)=\gamma T+\frac{1}{2}\lambda \sum_{i=0}^{T}w_{0}^{2}$. *T* is the number of leaf nodes, $w_{j}$ is the output of leaf node *j*, *λ* is the number of leaf nodes, and *γ* is the regularization parameter of the output. The model structure can be represented as shown in Figure 6.

### 4.3. SVR Model

Support Vector Machine (SVM) is a machine learning algorithm that determines the values of the parameters w and b by training the model on samples to finally obtain a regression function of the form $f\left(x\right)=\omega^{T}x+b$ [26]. In this experiment, for the nonlinear data, the samples first need to be linearly separated in a higher-dimensional space by nonlinear mapping. At this point, the computational volume will be explosive, and a kernel function needs to be introduced to reduce the computational complexity [27]. With the introduction of the kernel function, the SVM’s nonlinear fitting function is as follows:

(9) $$f\left(x\right)=\omega^{T}x+b=\overset{n}{\underset{i=1}{\sum }}\left(a_{i}-a_{i}^{*}\right)K\left(x_{i},x_{j}\right)+b$$

where $K\left(x_{i},x_{j}\right)$ is the kernel function, which represents the inner product operation of the mapping results of the input eigenvalues of the samples *i* and *j*; $a_{i}$ and $a_{i}^{*}$ are Lagrangian multipliers.

SVR is an important application branch of SVM and is a model of SVM applied to regression problems. Moreover, the SVR model has many variants based on different kernel functions [28]. Since the problem to be solved in this study had no previous research to refer to, after several attempts, we finally selected the Gaussian kernel function with the best results for the prediction of the model. The comparison results of the kernel functions we adopted are shown in Table 2.

### 4.4. BPNN

The BPNN is divided into two propagation processes: forward and reverse. To avoid overfitting due to excessive complexity, a single implicit layer was used and the number of nodes in the implicit layer was set to 8 through several experiments. Adam was chosen as the optimizer and 3-fold cross-validation was used. The grid search function GridSearchCV was used to optimize the super-parameters of the model. The hidden layer used Tansigs-type functions and the model was trained using the Levenberg–Marquardt back-propagation algorithm [29,30]. After the forward propagation of each input to the output layer, the error back-propagation training corrected the weights and thresholds. The weights and thresholds were adjusted in several iterations until the errors were within the set requirements [31].

**Figure 7:**
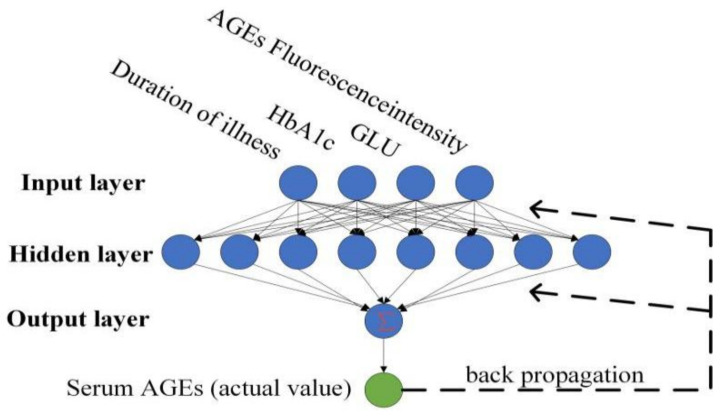
The BPNN model used for prediction.

An overall representation of the BPNN model is shown in Figure 7.

## 5. Results and Analysis

Single-input features and four-input features were added into the four modeling algorithms, and the two datasets were randomly divided into a training set and a test set at the ratio of 7:3, with 58 samples in the training set and 25 samples in the test set. Table 3 shows a comparison of the single-input and four-input prediction effects of the four models. The deviation between the real value and the model’s predicted value, and the goodness of the model’s predictive effect, were used as evaluation indices to investigate the mean square error (MSE) and the coefficient of determination (*R^2^*) of the final results. The predictions using single-input features in this dataset were better than those with four-input features, indicating that the introduction of the three selected features would add noise effects to the model [32]. This is mainly because the correlation between AGEs’ fluorescence intensity and the concentration of corneal AGEs is too high. Therefore, the final results of these four quantitative prediction models with single-input features were compared and analyzed next.

With a single-input feature, the MLR prediction model had the highest average error rate (AER) of 2.96%, while the SVR prediction model had the lowest AER of 2.43%. In addition, SVR had the best performance for both MSE and R-squared (*R^2^*).

**Figure 8:**
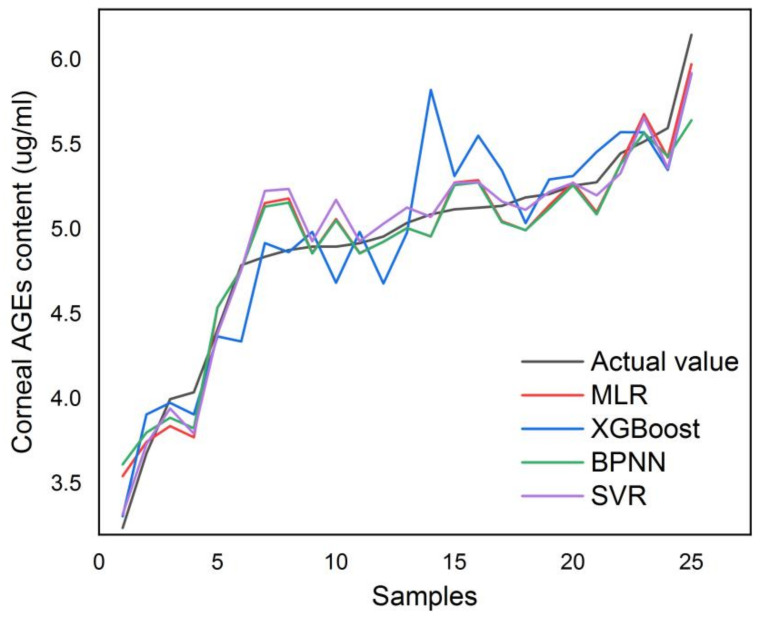
Comparison of the predictions.

Figure 8 shows a comparison of the predictions for the single-input case, and it can be intuitively seen that the predictions of SVR were closest to the true values. In summary, the SVR prediction model had better predictions in this work and was more suitable for modeling this dataset.

## 6. Conclusions

In this study, we designed a device to measure corneal AGEs based on the fluorescent properties of AGEs. To build an effective regression model to measure the concentration of human AGEs, we tested the performance of four machine learning models (SVR, BP, XGBoost, and MLR). Furthermore, we recruited 83 volunteers for clinical trials to obtain real data. The experimental results showed that the predictive effects of the model with a single-input feature were better than those with four inputs because the three features of disease duration, HbA1c, and GLU added noise to the model. In conclusion, the SVR prediction model had the best predictions among the four models developed with this dataset. With the SVR model, an AER of 2.43%, an MSE of 0.026, and an *R^2^* of 0.932 were obtained.

In further research, the range of test points should be widened to obtain a larger sample to improve the reliability of the test. In addition, the modeling algorithms used in this study all have hyperparameters that need to be set artificially, so heuristic parameter search methods, such as genetic algorithms and Particle Swarm Optimization, could be tried in future studies to perform global optimization in a larger search range to find the best prediction of the model. The portable fluorometer constructed in this study for the detection of AGEs provides a convenient method of immediate detection and has long-term monitoring potential for diabetic patients and pre-diabetic populations.

## Figures and Tables

**Table 1:** Physiological parameters and data on the fluorescence intensity of corneal AGEs.

Parameters	Diabetic Group	Healthy Group
Age (years)	53 ± 15	44 ± 13
Height (cm)	164.1 ± 6.5	162.4 ± 6.1
Weight (kg)	63.4 ± 7.2	56.7 ± 6.5
BMI (kg/m^2^)	23.51 ± 2.16	21.48 ± 1.72
Heart rate (beats/min)	76 ± 10	81 ± 8
Systolic pressure (mmHg)	131.50 ± 12.61	128.10 ± 6.62
Diastolic pressure (mmHg)	79.82 ± 8.30	80.10 ± 4.50
HbA1c (%)	8.30 ± 1.96	5.08 ± 0.37
GLU (mmol/L)	9.33 ± 2.27	5.25 ± 0.49
Fluorescence intensity of corneal AGEs	23.80 ± 2.12	13.27 ± 1.65

**Table 2:** Model effects under different kernel functions.

Kernel	Linear	Poly	Rbf
$R^{2}$	0.926	0.926	0.932
MSE	0.028	0.028	0.026

**Table 3:** Comparison of the predictive effects.

Evaluation Indices	MLR		XGBoost		SVR		BPNN	
	Single Input	Four Inputs	Single Input	Four Inputs	Single Input	Four Inputs	Single Input	Four Inputs
MSE	0.027	0.054	0.06	0.050	0.026	0.065	0.0283	0.075
*R^2^*	0.928	0.900	0.928	0.930	0.932	0.808	0.925	0.779
AER	2.96%	3.51%	2.73%	3.42%	2.43%	3.68%	2.90%	3.82%

## Data Availability

The source codes related to the present work can be found at: https://github.com/23Air/algorithnm.git (accessed on 9 December 2022).
